# Diaspididae (Hemiptera: Coccoidea) of Espírito Santo, Brazil

**DOI:** 10.1673/031.008.1701

**Published:** 2008-03-05

**Authors:** Mark P. Culik, David S. Martins, José A. Ventura, Vera S. Wolff

**Affiliations:** ^1^ Institute Capixaba de Pesquisa, Assistência Técnica e Extensão Rural - INCAPER, Rua Afonso Sarlo 160, CEP 29052-010, Vitória, Espírito Santo, Brasil; ^2^ Fundação Estadual de Pesquisa Agropecuária FEPAGRO, Rua Gonçalves Dias, 570, (Menino Deus), 90130-060, Porto Alegre, RS, Brasil

**Keywords:** armored scale insects, biodiversity, biogeography, host plants

## Abstract

Twenty-seven species of armored scale insects (Hemiptera: Diaspididae) are newly recorded from Espírito Santo, Brazil, and information on the host plants and geographic distribution of the 31 species of Diaspididae that have been identified in the State is provided. New plant host records are reported for 11 of the diaspidid species studied and results are discussed with respect to development of agriculture in this and similar areas with objectives of modernization and diversification.

## Resumo

São registradas pela primeira vez no Estado de Espírito Santo, Brasil, vinte e sete espécies de cochonilhas da família Diaspididae (Hemiptera: Coccoidea), com informações sobre as plantas hospedeiras e a distribuição geográfica das 31 espécies que já haviam sido identificadas no Estado. São registradas novas plantas hospedeiras de 11 espécies de cochonilhas e são discutidos os resultados com respeito ao desenvolvimento da agricultura no Estado e áreas similares com objetivos de modernização e diversificação.

## Introduction

Armored scales (Hemiptera: Diaspididae) are sap feeding insects that are important pests of many agricultural crops and ornamentals throughout the world because of the damage that they cause to plants. In addition, because it is often difficult to remove these insects from produce such as fruits they may be considered to be cosmetically damaging and many diaspidid species are also of quarantine concern requiring management to prevent their spread through export of plant products (Miller et al. 2005).

Although approximately 150 diaspidid species have been recorded from Brazil, only 5 have been previously identified from the State of Espírito Santo (Silva et al. 1968; Claps et al. 1999; Claps et al. 2001; Martins et al. 2004). Espírito Santo is a relatively small State (46,078 km^2^) located in the east central region of Brazil (between ∼18°S and 21°S) but includes a diverse variety of environments ranging from coastal to ∼3,000 m altitude. The State is at the center of one of the world's most biologically diverse ecosystems, the Mata Atlântica, and contains some of the most biologically diverse forests in the world (Mori 1989; Thomaz & Monteiro 1997). Agriculture is also an important part of the State's economy with major crops including coffee, sugarcane, and fruits such as papaya. Preservation of biodiversity and development of sustainable agriculture, based on practices such as integrated pest management (IPM), in Espírito Santo and similar areas depends on increased knowledge of the actual biological diversity (pest and beneficial insects, for example) present in such areas. Therefore, because information on the insect fauna of Espírito Santo is needed for preservation of biodiversity and sustainable development in this State, scale insects were collected during 2003 to 2006 from various plants in the State to identify the species present in this area. Here we document new armored scale insect records for Espírito Santo based on these recent collections to serve as a reference to the known scale insect fauna of this area, as well as contribute to a more complete knowledge of diaspidid biogeography and host plant relationships in general.

## Materials and Methods

Diaspidids were collected during surveys of the insect fauna of papaya and pineapple in Espírito Santo and when noticed on plants during fieldwork or other activities in 2003 to 2007.

Samples of plant parts (fruits, leaves, stems) infested with diaspidids were collected from locations throughout the State ranging from municipalities of Pinheiros in the north (18.40°S; 40.21°W) to Marataízes in the south (21.03°S; 40.83°W) and Vitória (20.32°S; 40.32°W) on the coast to municipalities in the interior of the State such as Venda Nova do Imigrante (20.38°S; 41.19°W), and from a variety of sites including experimental research plots, commercial fields, private homes, and the Reserva Natural da Vale do Rio Doce. The samples were transported to the Espírito Santo rural research and extension institute INCAPER (Institute Capixaba de Pesquisa, Assistência Técnica e Extensão Rural) headquarters in Vitória for photographing and preservation of the diaspidid specimens. The specimens were slide-mounted for identification using 10% sodium hydroxide for clearing, dehydration in alcohol, and Canada balsam mounting medium. Voucher specimens of these insects are deposited in the arthropod collections of INCAPER, Vitória, Espírito Santo, and the Museu de Entomologia Professor Ramiro Gomes Costa, Fundação Estadual de Pesquisa Agropecuária FEPAGRO, Porto Alegre, Rio Grande do Sul, Brasil.

## Results

**Figure 1.**
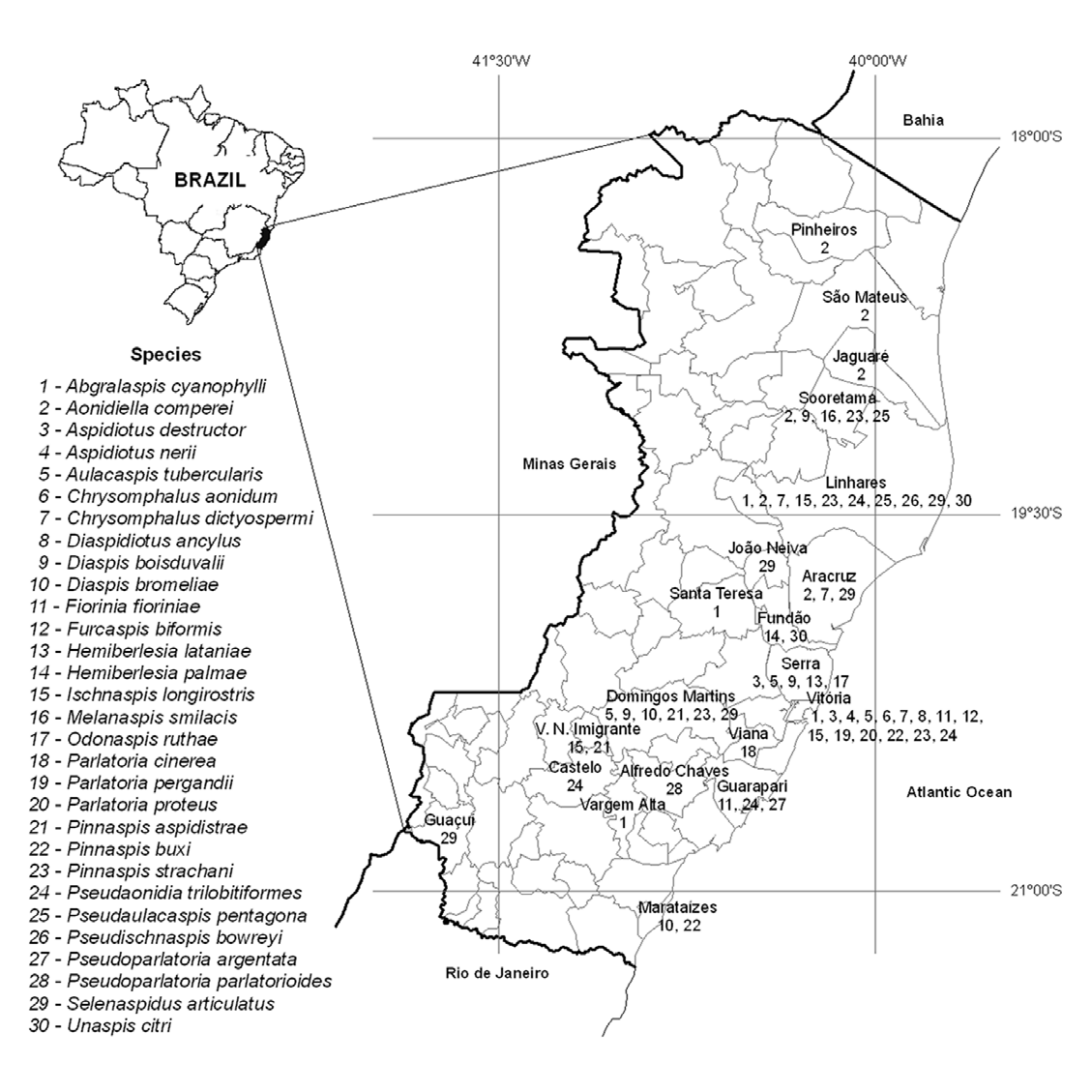
Map of the State of Espírito Santo, Brazil, showing municipalities where Diaspididae species (1–30) were collected in this study, 2003–2007. Note: The name of the species 24 is misspelled. It should be *Pseudaonidia trilobitiformis.*

Diaspidids were identified from approximately 100 plant samples representing at least 30 species in 20 plant families consisting mainly of tropical fruits and ornamentals. Twenty-seven species of armored scale insects that have not previously been recorded from Espírito Santo were identified in this study, bringing the total number of species of armored scale insects known from this State to 31 (Table 1, Figure 1). *Lepidosaphes gloverii* (Packard) was incorrectly reported from Espírito Santo (and several other states in Brazil) in Claps et al. (2001). The known geographic distribution of *L. gloverii* in Brazil is São Paulo (Silva *et. al* 1968) and Rio Grande do Sul (Wolff and Corseuil 1994). Most of the diaspidids identified from Espírito Santo are known to have a broad plant host range and wide geographic distribution. However, 11 of the scale species that were collected were found on new host plants in this study, most notably *Diaspidiotus ancylus* (Putnam) on a new host family, *Psidium guajava* (Myrtaceae).

Most of the diaspidids that have been identified from Espírito Santo are also potential pests of a variety of economically important crops in the State, for example, citrus, mango, and coconut. It is especially notable that four potential pests of coffee: *Diaspis boisduvalii* Signoret, *Parlatoria proteus* (Curtis), *Pseudaonidia trilobitiformis* (Green), and *Selenaspidus articulatus* (Morgan); seven potential pests of papaya: *Aspidiotus destructor* Signoret, *Aspidiotus nerii* Bouche, *Chrysomphalus dictyospermi* (Morgan), *P. trilobitiformis, Pseudaulacaspis pentagona* (Targioni Tozzetti), *Pseudoparlatoria parlatorioides* (Comstock), and *S. articulatus;* and seven potential pests of pineapple: *A. nerii, D. boisduvalii, D. bromeliae* (Kerner), *Melanaspis smilacis* (Comstock), *Pinnaspis strachani* (Cooley), *P. trilobitiformis,* and *Unaspis citri* (Comstock), are recorded here for the first time to be present in the State (Table 1).

## Discussion

At least 35 additional species of Diaspididae are known from States neighboring Espírito Santo; Bahia, Minas Gerais, and Rio de Janeiro (Claps et al. 1999; Claps et al. 2001). Thus, it is likely that many more diaspidid species are actually present in Espírito Santo and likely to be found with additional study. Although about half of the ∼200 diaspidid species known from the region of Brazil, Chile and Argentina, are considered to be exotic (Claps et al. 1999; Claps et al. 2001), it is also of interest to note that almost all of the diaspidids currently known from Espírito Santo are considered to be exotic to the region (Claps et al. 1999, 2001), and of those whose origin has been proposed, most (at least 75%) are believed to be of non-neotropical origin (Watson 2006), perhaps indicating the dangers of the potential dominance of introduced species in areas such as Espírito Santo.

**Table 1.**
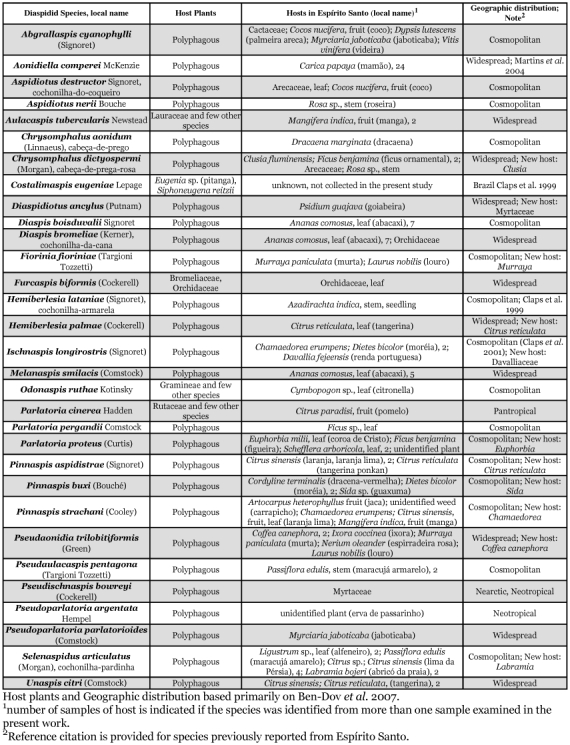
Diaspididae of Espírito Santo (ES), Brazil: this study (2003–2006) and previous records

Few species of diaspidid scale insects have previously been noted as pests in Espírito Santo.

However, most of the species of Diaspididae identified in this study are polyphagous and potential pests of many crops (Table 1, and Ben-Dov et al. 2006). And, agriculture in Espírito Santo is currently undergoing a major transition (diversification) from an agriculture dominated by coffee production to increased production of a diverse variety of high value crops such as papaya and other fruits (Alves 2003). Improper use of pesticides may suppress natural enemies and lead to outbreaks of diaspidid pests (Raupp et al. 2001). Natural enemies of scale insects, such as species of *Aphytis* and *Encarsia* that are parasitoids *of Aonidiella comperei* Mckenzie, are present in Espírito Santo (Marangoanha et al. 2005) and it is likely that a reason relatively few diaspidids have previously been identified as pests in this State is because natural enemies have been effective in maintaining scale insect populations below noticeable (or economically damaging) levels. Therefore, although most of the diaspidids identified in this study are not currently known as major pests in Espírito Santo, recognition of the presence of these potential pests should be considered in development of agriculture in the State to avoid practices, such as the misuse of pesticides and destruction of natural enemies, that may favor development of these insects as pests in the future.

Just as information on the insects present in an area is essential as a basis for any rational, sustainable management of agricultural pests, knowledge of the actual biological diversity present in specific areas is essential for preservation of biodiversity. As pointed out by Staube (2004), knowing what species occur in a specific region is the only means of determining its biodiversity. Such faunistic information is the basic material required for studies of biogeography which in turn are essential for conservation (Staube 2004). Unfortunately, there is a lack of recognition of the importance of this type of information by many scientists and lack of support for its publication (Straube 2004). Such problems and the need for information on the world's biodiversity have also been noted by Wilson (2000) and Valdecasas and Camacho (2003) among others. This faunistic information is essential for biogeography, conservation, and taxonomy, as well as for pest management.

Results of this study confirm that a diverse diaspidid, potential pest, fauna is present in Espírito Santo and indicate the need for researchers and producers to develop and utilize integrated pest management methods to avoid practices that may favor the development of these potential pests in the future. Accurate information on the insects present in an area is essential as a basis for development of integrated pest management and this information on the armored scales present in Espírito Santo should better enable researchers and producers to develop and utilize integrated pest management practices in this State. Actual knowledge of the biological diversity present in areas such as Espírito Santo is also essential for preservation of biodiversity and these results are a contribution to such knowledge. The armored scale insect records for Espírito Santo documented here should also serve as a basic reference to the known scale insect fauna of this area, as well as contribute to a more complete knowledge of diaspidid biogeography and host plant relationships in general.

Paper copies of this article will be deposited in the following libraries. Senckenberg Library, Frankfurt Germany; National Museum of Natural History, Paris, France; Field Museum of Natural History, Chicago, Illinois USA; the University of Wisconsin, Madison, USA; the University of Arizona, Tucson, Arizona USA; Smithsonian Institution Libraries, Washington D.C. U.S.A.; The Linnean Society, London, England.
